# Fatty Acid Oxidation and Mitochondrial Morphology Changes as Key Modulators of the Affinity for ADP in Rat Heart Mitochondria

**DOI:** 10.3390/cells9020340

**Published:** 2020-02-01

**Authors:** Adolfas Toleikis, Sonata Trumbeckaite, Julius Liobikas, Neringa Pauziene, Lolita Kursvietiene, Dalia M. Kopustinskiene

**Affiliations:** 1 Neuroscience Institute, Lithuanian University of Health Sciences, Eiveniu 4, LT-50161 Kaunas, Lithuania; tadolfas@yahoo.co.uk (A.T.); Sonata.Trumbeckaite@lsmuni.lt (S.T.); Julius.Liobikas@lsmuni.lt (J.L.); 2 Department of Pharmacognosy, Medical Academy, Lithuanian University of Health Sciences, Sukileliu pr. 13, LT-50166 Kaunas, Lithuania; 3 Department of Biochemistry, Medical Academy, Lithuanian University of Health Sciences, Eiveniu 4, LT-50161 Kaunas, Lithuania; Lolita.Kursvietiene@lsmuni.lt; 4 Institute of Anatomy, Lithuanian University of Health Sciences, Mickeviciaus 9, LT-44307 Kaunas, Lithuania; Neringa.Pauziene@lsmuni.lt; 5 Institute of Pharmaceutical Technologies, Medical Academy, Lithuanian University of Health Sciences, Sukileliu pr. 13, LT-50161 Kaunas, Lithuania

**Keywords:** ADP/ATP carrier, K_m_^ADP^, mitochondria, fatty acid oxidation, dextran, morphology

## Abstract

Fatty acids are the main respiratory substrates important for cardiac function, and their oxidation is altered during various chronic disorders. We investigated the mechanism of fatty acid–oxidation-induced changes and their relations with mitochondrial morphology and ADP/ATP carrier conformation on the kinetics of the regulation of mitochondrial respiration in rat skinned cardiac fibers. Saturated and unsaturated, activated and not activated, long and medium chain, fatty acids similarly decreased the apparent K_m_^ADP^. Addition of 5% dextran T-70 to mimic the oncotic pressure of the cellular cytoplasm markedly increased the low apparent K_m_^ADP^ value of mitochondria in cardiac fibers respiring on palmitoyl-l-carnitine or octanoyl-l-carnitine, but did not affect the high apparent K_m_^ADP^ of mitochondria respiring on pyruvate and malate. Electron microscopy revealed that palmitoyl-l-carnitine oxidation-induced changes in the mitochondrial ultrastructure (preventable by dextran) are similar to those induced by carboxyatractyloside. Our data suggest that a fatty acid oxidation-induced conformational change of the adenosine diphosphate (ADP)/adenosine triphosphate (ATP) carrier (M-state to C-state, condensed to orthodox mitochondria) may affect the oxidative phosphorylation affinity for ADP.

## 1. Introduction

The major part of energy supply in cells comes from the fatty acid oxidation in mitochondria. Fatty acids as the main respiratory substrates are important, not only for cardiac function [1,2], but also they recently have been shown to provide energy for the proliferation and survival of tumors [3]. An increased fatty acid consumption reduces cardiac efficiency, and among the mechanisms involved, a modulation of the ADP/ATP carrier has been suggested [4,5,6].

The studies of saponin-permeabilized heart and skeletal muscle fibers demonstrated that, in contrast to isolated mitochondria, the mitochondrial outer membrane in situ possesses a low permeability for exogenous ADP (high apparent K_m_^ADP^ of oxidative phosphorylation for external ADP), and therefore, is crucial in the mechanism of regulation of mitochondrial respiration in vivo [7,8]. K_m_^ADP^ is drastically (up to 10-fold) decreased in the case of the oxidation of saturated fatty acids (CoA- or carnitine-esters of palmitate and octanoate; alone or in combination with pyruvate), but not during the transport of fatty acids into mitochondria [9]; however, the mechanism of this phenomenon has not been elucidated yet.

Mitochondria and intracellular ATPases in cardiomyocytes are in close proximity, and are arranged into tightly coupled structural and functional complexes known as intracellular, energetic units [8,10,11]. Strict interpositions of mitochondrion optimizes the energy fluxes and interactions of mitochondria with surrounding organelles; however, at high workload the direct ATP transfer does not fulfil the energy need in heart cells [12,13]. In these conditions, the creatine kinase–phosphocreatine system is useful: creatine (the substrate of mitochondrial creatine kinase) significantly stimulates the production of ADP (when the concentration of ADP is suboptimal, i.e., about 50–10 µM) by mitochondrial creatine kinase functionally coupled with the ADP/ATP carrier. This significantly enhances the respiration of heart mitochondria and ATP production. The stimulating effect of creatine on respiration decreases when the permeability of the mitochondrial outer membrane for ADP increases (the apparent K_m_^ADP^ decreases) due to the treatment of cardiac cells with proteolytic enzymes (trypsin or collagenase) [14,15], after the isolation of mitochondria [16,17], or due to some clustering of mitochondria possessing an intact mitochondrial outer membrane and the alteration of their position within the cells of the non-ischemic zone of the low-flow-perfused rat heart [18]. 

Fatty acids have been demonstrated to change the conformation of uncoupling protein [19]. Furthermore, other studies revealed structural, and to some extent functional, homology between the uncoupling protein and the ADP/ATP carrier [20,21]. There is also an indirect evidence that low apparent K_m_^ADP^ in mitochondria from cancerous cells [22,23] and fetal or neonatal mitochondria [24] could be related to mitochondrial ultrastructural changes, namely increased, diffuse the mitochondrial matrix volume corresponding to orthodox mitochondrial conformation [25,26].

Mitochondria are significantly more abundant in hearts compared to skeletal muscle, brain, kidney and liver [27]. Furthermore, cardiac mitochondria primarily use fatty acids as respiratory substrates, whereas most other organs use glucose as the major energy substrate [28], and therefore, we have chosen skinned cardiac fibers as the experimental object in our study. We investigated the mechanism of fatty acid–oxidation-induced changes and their relations with mitochondrial morphology and ADP/ATP carrier conformation on the kinetics of the regulation of mitochondrial respiration (the apparent K_m_^ADP^) in situ; i.e., in the mitochondria of saponin-permeabilized rat heart muscle fibers, where mitochondria and the ATPases in myofibrils and in the sarcoplasmic reticulum remain intact, corresponding to the physiological conditions in the cell [8,10].

## 2. Materials and Methods

All chemicals used in this work were from Sigma-Aldrich (St. Louis, MO, USA).

The male Wistar rats ~3 months old and weighing 250–300 g were obtained from the Vivarium of the Lithuanian University of Health Sciences, where they were housed at 23 ± 2 °C with a 12-h light/dark cycle and free access to food and water. The experimental procedures used in the present study were performed according to the permission of the Lithuanian Committee of Good Laboratory Animal Use Practice (number 0228/2012). Rats were killed by cervical dislocation. Rat hearts were excised and rinsed in ice-cold 0.9% KCl solution. The bundles of cardiac fibers, approximately 0.3–0.4 mm in diameter, were prepared by using sharp-ended needles from the muscle strips cut out from the left ventricular endocardium in an ice-cold preparation solution containing 20 mM imidazole, 20 mM taurine, 0.5 mM dithiothreitol, 7.1 mM MgCl_2_, 50 mM 2-(NMorpholino)ethanesulfonic acid (MES), 5 mM adenosine triphosphate (ATP), 15 mM phosphocreatine, 2.62 mM CaK_2_EGTA and 7.38 mM K_2_EGTA (ionic strength of the solution 160 mM, free Ca^2+^ 0.1 μM, free Mg^2+^ 3 mM; pH 7.0, adjusted with KOH), then permeabilized by saponin (50 μg/mL, 30 min), washed for 10 min in a physiological salt solution containing 20 mM imidazole, 20 mM taurine, 0.5 mM dithiothreitol, 1.61 mM MgCl_2_, 100 mM MES, 3 mM KH_2_PO_4_, 2.95 mM CaK_2_EGTA and 7.05 mM K_2_EGTA (ionic strength of the solution 160 mM, free Ca^2+^ 0.1 μM, free Mg^2+^ 1 mM; pH 7.1, adjusted with KOH) [29]. All procedures were carried out under intensive shaking (120 times/min). The washed bundles of fibers were rinsed once in the physiological salt solution, transferred into the tubes with the same solution and then kept on ice.

Respiration rates of skinned cardiac fibers were determined in the closed respiration chamber in physiological salt solution at 37 °C or 25 °C by the means of the Clark-type oxygen electrode. Pyruvate + malate (6 mM + 6 mM), glutamate 6 mM + malate (6 mM + 6 mM), palmitoyl-l-carnitine+malate (9 μM + 0.24 mM), oleoyl-CoA + l-carnitine + malate (6 µM + 2.5 mM + 0.24 mM) or decanoic acid + pyruvate + malate (0.3 mM + 6 mM + 6 mM) were used as respiratory substrates as indicated in the Results section or in the Figure legends. Respiration rates were expressed as nmol O/min/mg fibers’ dry weight. Dry weight = wet weight before respiration measurement/factor ‘*W*’. The factor ‘*W*’ was calculated to be 4.85 for heart muscle fibers [14]. The solubility of oxygen was taken to be 422 nmol O/mL at 37 °C and 452 nmol O/mL at 25 °C [30]. The adenosine diphosphate (ADP) regenerative system, consisting of 1.2 IU/mL lyophilized yeast hexokinase (Type V; EC 2.7.1.1) and 24 mM glucose, was added to the chamber before the addition of heart muscle fibers. Titration was made by different ADP concentrations in each separate probe. ΔV was expressed as a difference between respiration rates in the presence and in the absence of added ADP. The apparent K_m_^ADP^ and V_max_ were estimated from the least-squares fit to the Michaelis–Menten equation (ΔV vs. ADP concentration). 

Mitochondria were isolated by a differential centrifugation procedure. Hearts were excised and rinsed in ice-cold isolation medium, containing 160 mM KCl, 10 mM NaCl, 20 mM Tris, 5 mM EGTA (pH 7.7, adjusted with KOH at 2 °C). Mitochondria were isolated in the same medium supplemented with 2 mg/mL bovine serum albumin (BSA). Homogenate was centrifugated for 5 min at 750× *g*, then the supernatant was centrifugated for 10 min at 6740× *g* and the pellet was washed once in the medium containing 180 mM KCl, 20 mM Tris, 3 mM EGTA (pH 7.3 adjusted with KOH at 2 °C), suspended in it and kept on ice. The mitochondrial protein concentration was determined by the biuret method (Piotrowski’s test) [31]. The final mitochondrial protein concentration was 0.5 mg/mL. Mitochondrial swelling was recorded as the decrease of light scattering at 540 nm with the Heλios α spectrophotometer in physiological salt solution supplemented with 24 mM glucose and 1.2 IU/mL hexokinase, 0.24 mM malate and palmitoyl-l-carnitine (9–80 µM).

An exogenous ADP-trapping system consisting of pyruvate kinase + phosphoenolpyruvate (PK + PEP), which effectively competes with mitochondria for the extramitochondrial ADP, and therefore, decreases the respiration rate in the State 3, was used to investigate the interactions of the functional complexes of mitochondria with Ca, the MgATPases of myofibrils and the sarcoplasmic reticulum under the different conditions (25 °C and 37 °C; mitochondria oxidizing different substrates: glutamate 6 mM + malate 6 mM or palmitoyl-l-carnitine 9 μM + malate 0.24 mM). The sequence of additions to the respiration chamber: 8 mM PEP, ~3 mg of cardiac fibers, 2 mM ATP, 20 + 20 U/mL (or 40 U/mL) PK, 20 mM creatine, 35 μM cytochrome c, 125 μΜ atractyloside. After each addition, the respiration rate was estimated. The cytochrome c test was used to evaluate the intactness of the mitochondrial outer membrane.

The coupling of mitochondrial creatine kinase (mi-CK) and the ADP/ATP carrier was estimated using two approaches. (1) The apparent K_m_^ADP^ and V_max_ values were estimated from ΔV vs. ADP concentration relationships in the presence or in the absence of 20 mM creatine; the results were compared with corresponding kinetic parameters without creatine; (2) 60 μM ADP was added into the respiration chamber followed by the addition of 20 mM of creatine. The stimulation of respiration by creatine, i.e., the creatine effect, was expressed as the ratio of the respiration rates with creatine and with 60 μM of ADP without creatine.

For the electron microscopy studies, the saponin-permeabilized rat cardiac fibers were incubated aerobically in the physiological salt solution containing pyruvate and malate, 6 mM both (without, as control, or with carboxyatractyloside 1.3 µM or bongkrekic acid 17.6 µM), or palmitoyl-l-carnitine 9 μM, or palmitoyl-l-carnitine 9 μM plus 5% dextran T-70, for 5 min at 37 °C under intensive stirring. Subsequently, the fibers were placed into 2.5% glutaraldehyde solution in 0.1 M cacodylate buffer (pH 7.4) and kept in it for 5 min at room temperature under gentle shaking. Afterwards, the fibers were left in the same fixative overnight at 4 °C. Later on, the fibers were washed several times in cacodylate buffer and post-fixed for 1 h at 4 °C with 1% osmium tetraoxide solution in the same buffer. After that, they were dehydrated through a graded ethanol series and embedded in a mixture of resins Epon 812 and Araldite. Ultrathin sections were cut with a with ultra-microtome, stained with uranyl acetate and lead citrate, and examined with a PHILIPS EM300 electron microscope, using AGFA electron image films.

The results are presented as means ± S.E. The data were analyzed with one-way analysis of variance (ANOVA) by Prism v. 5.04 (GraphPad Software Inc., La Jolla, CA, USA). Then *p* <0.05 was taken as the level of significance.

## 3. Results

### 3.1. Role of Oxidation of Fatty Acids in Regulation of Oxidative Phosphorylation and Mitochondrial Swelling

**Figure 1:**
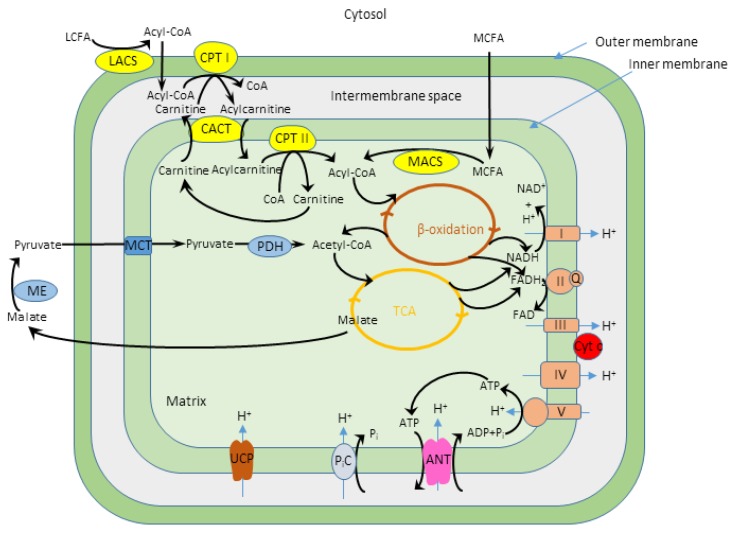
The principal scheme of the fatty acid and pyruvate and malate oxidation in mitochondria. LCFA—long chain fatty acids, MCFA—medium chain fatty acids, LACS—long chain acyl-CoA synthetase, CPT—carnitine palmitoyl-transferase, CACT—carnitine acylcarnitine translocase, MACS—medium chain acyl-CoA synthetase, ME—malic enzyme, MCT—monocarboxylate transporter, PDH—pyruvate dehydrogenase, TCA—tricarboxylic acid cycle, NAD—nicotinamide adenine dinucleotide, FAD—flavin adenine dinucleotide, I-V—mitochondrial electron transport chain complexes, Q—coenzyme Q, Cyt c—cytochrome c, ANT—ADP/ATP carrier, P_i_C—inorganic phosphate carrier, UCP—uncoupling protein.

Oxidation of fatty acids, the major myocardial respiratory substrates (palmitoyl-l-carnitine, palmitoyl-CoA + l-carnitine and octanoyl-l-carnitine) caused the drastic decrease of the apparent K_m_^ADP^ specific for pyruvate+malate oxidation [9], but the mechanisms of this effect have not been elucidated yet. In this study, we investigated which factors are responsible for the low apparent K_m_^ADP^ observed during fatty acid oxidation. Firstly, we evaluated if the fatty acid related decrease of the apparent K_m_^ADP^ depend on the different fatty acid chain length and degree of saturation. The principal scheme of fatty acid and pyruvate and malate oxidation in mitochondria is presented in Figure 1.

**Figure 2:**
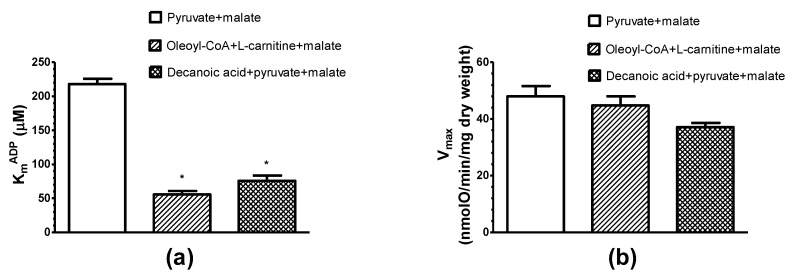
Influence of different respiratory substrates: pyruvate + malate (6 mM + 6 mM), oleoyl-CoA + l-carnitine+malate (6 µM + 2.5mM + 0.24mM) and decanoic acid + pyruvate + malate (0.3 mM + 6 mM + 6 mM) on the apparent K_m_^ADP^ (**a**) and V_max_ (**b**) of saponin-permeabilized rat cardiac fibers. *n* = 5; 37 °C; * *p* < 0.05 vs. control (pyruvate + malate). The results were analyzed with one-way analysis of variance (ANOVA) followed by the Dunnett post hoc test.

In the case of pyruvate+malate oxidation the apparent K_m_^ADP^ of mitochondria in saponin-permeabilized rat cardiac fibers (K_m_^ADP^ = 217.8 ± 8 µM) is by 3–10 folds higher if compared with the apparent K_m_^ADP^ for oleoyl-CoA + l-carnitine+malate (55.7 ± 5.1 µM ADP), decanoic acid + pyruvate + malate (75.8 ± 7.6 µM ADP) or isolated rat heart mitochondria (K_m_^ADP^ = 23 µM, Liobikas et al., 2001) (Figure 2). It is evident that the oxidation of both decanoic acid and oleoyl-CoA in situ induces a marked increase in the affinity of mitochondrial oxidative phosphorylation system for ADP.

**Figure 3:**
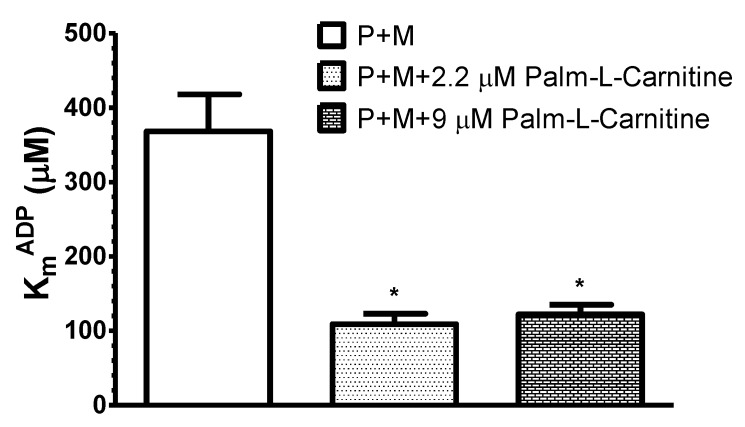
Influence of different respiratory substrates: pyruvate + malate (P + M, 6 mM + 6 mM), pyruvate + malate + palmitoyl-l-carnitine (6 mM + 6 mM + 2.2 µM) and pyruvate + malate + palmitoyl-l-carnitine (6 mM + 6 mM + 9 µM) on the apparent K_m_^ADP^ of saponin-permeabilized rat cardiac fibers. Here, *n* = 5; 37 °C; * *p* < 0.05 vs. control (pyruvate + malate). The results were analyzed with one-way analysis of variance (ANOVA) followed by the Dunnett post hoc test.

In the next series of experiments on different skinned cardiac fiber samples we tested the concentration dependence of the effect of fatty acids on the apparent K_m_^ADP^ (Figure 3). The high apparent K_m_^ADP^ with pyruvate + malate decreased to 30% and 33% of control values, when pyruvate + malate was supplemented with 2.2 μM or 9 μM palmitoyl-l-carnitine. 

**Figure 4:**
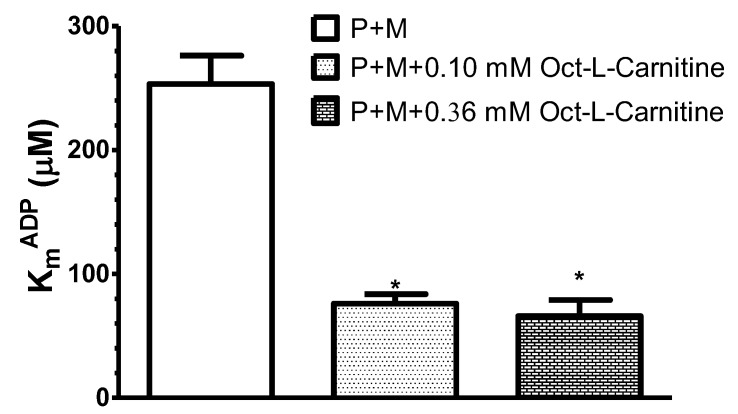
Influence of different respiratory substrates: pyruvate + malate (P + M, 6 mM + 6 mM), pyruvate + malate + octanoyl-l-carnitine (6 mM + 6 mM + 0.36 mM) and pyruvate + malate + octanoyl-l-carnitine (6 mM + 6 mM + 0.1 mM) on the apparent K_m_^ADP^ of saponin-permeabilized rat cardiac fibers. In this case, *n* = 4; 37 °C; * *p* < 0.05 vs. control (pyruvate + malate). The results were analyzed with one-way analysis of variance (ANOVA) followed by the Dunnett post hoc test.

Thus, four times lower concentration of palmitoyl-l-carnitine (2.2 μM) also effectively decreased the apparent K_m_^ADP^. Noteworthy, at 2.2 μM palmitoyl–l-carnitine (alone) the State 3 respiration rate was equal to 30–40% of that estimated at 9 μM. In the separate set of experiments, we revealed also that even 0.5 µM of palmitoyl-l-carnitine (used together with pyruvate+malate) decreased the apparent K_m_^ADP^ (*p* < 0.05) to similar level as 9 µM of palmitoyl-l-carnitine (94.6 ± 13 µM and 77.9 ± 11 µM, respectively, compared with pyruvate+malate alone, 253.3 ± 23 µM; data of 4–6 paired experiments; 37 °C). Essentially similar results were obtained in the separate group of experiments when octanoyl-l-carnitine at two different concentrations (0.36 and 0.1 mM) was used as respiratory substrate in the presence of pyruvate + malate (Figure 4).

In this case, the apparent K_m_^ADP^ decreased, respectively, to 34% and 26% of control values estimated with pyruvate+malate (*p* < 0.05, *n* = 4, 37 °C). Thus, our results showed that the decrease in the apparent K_m_^ADP^ value did not depend upon the concentration of fatty acids.

**Figure 5:**
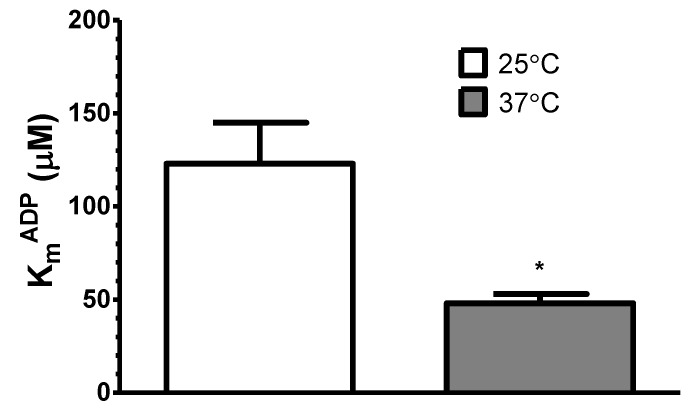
Influence of temperature on the apparent K_m_^ADP^ of saponin-permeabilized rat cardiac fibers oxidizing palmitoyl-l-carnitine (9 μM) in the presence of malate (0.24 mM). The results were analyzed with paired t test; *n* = 7; * *p* < 0.05.

Interestingly, during palmitoyl-l-carnitine oxidation, the apparent K_m_^ADP^ decreased by about 2.6-fold with elevation of temperature from 25 °C to 37 °C, i.e., from 123 ± 22 µM ADP to 48 ± 5 µM ADP, respectively (Figure 5).

**Figure 6:**
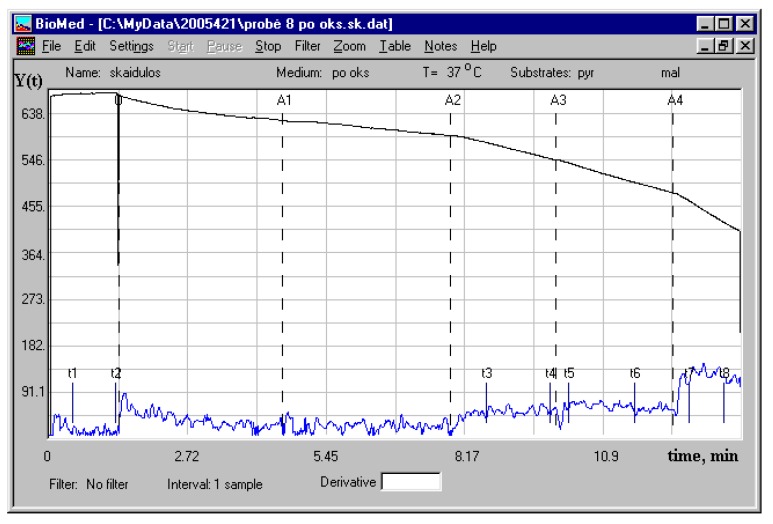
Representative respiration curve after the complete oxidation of a limited quantity of palmitoyl-l-carnitine. The black curve—oxygen consumption, the blue curve—oxygen consumption derivative. Additions: O—skinned rat cardiac fibers (registration of respiration on endogenous substrates), then A1—1.6 nmol of palmitoyl-l-carnitine, A2—pyruvate + malate (6 mM + 6 mM, respiration rate: 24.8 nmol/O/min/mg dry weight), A3—10 µM ADP (respiration rate: 32 nmol/O/min/mg dry weight), A4—1.2 mM ADP (respiration rate: 57.7 nmol/O/min/mg dry weight).

When pyruvate+malate were added immediately after the complete oxidation of a limited quantity (1.6 nmol) of palmitoyl-l-carnitine (Figure 6), the apparent K_m_^ADP^ of mitochondria in cardiac fibers remained at a high level (211 ± 40 µM ADP), similar as in the case of pyruvate+malate oxidation alone (223 ± 56 µM ADP) (data of five paired experiments, 37 °C). Thus, the effect of fatty acids on the apparent K_m_^ADP^ of mitochondria in permeabilized rat cardiac fibers is reversible.

**Figure 7:**
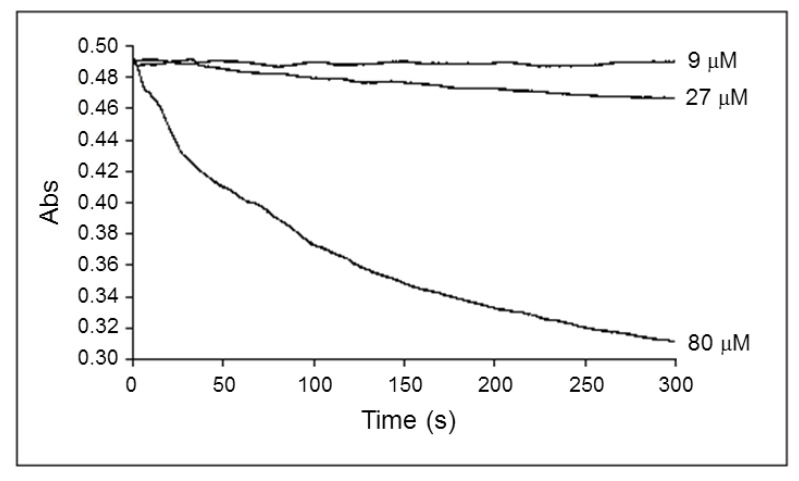
Swelling of isolated rat heart mitochondria respiring on palmitoyl-l-carnitine (9–80 µM) and malate (0.24 mM). *n* = 3, typical absorption traces are shown.

To exclude that the decrease in the apparent K_m_^ADP^ during fatty acid oxidation is related to the detergent activity of palmitoyl-l-carnitine (reviewed in Goni FM et al. 1966) and its damage to mitochondrial membranes, we measured the swelling of isolated rat heart mitochondria oxidizing palmitoyl-l-carnitine (9, 27 and 80 µM) and malate (0.24 mM). Our data (Figure 7) showed that palmitoyl-l-carnitine at a concentration of 9 μM did not induce mitochondrial swelling.

However, the higher concentrations (starting from 27 μM) of a palmitoyl-l-carnitine-induced concentration–dependent increase in mitochondrial swelling. Importantly, palmitoyl-l-carnitine did not change the mitochondrial outer membrane integrity (external cytochrome c did not stimulate the State 3 respiration; its effect varied between 0.98 and 1.02 at distinct palmitoyl-l-carnitine concentrations; *n* = 8).

### 3.2. Regulation of Mitochondrial Respiration by Endogenous versus Exogenous ADP: Influence of Palmitoyl-l-Carnitine

**Figure 8:**
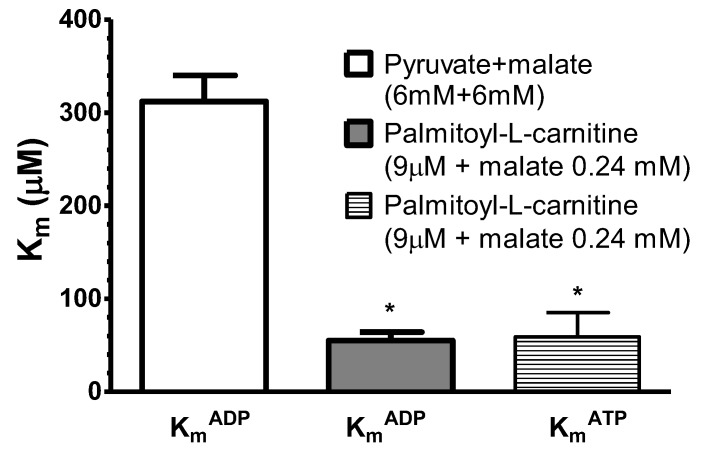
Influence of the adenosine diphosphate (ADP) source (endogenous (from exogenous adenosine triphosphate (ATP)) or exogenous) on the apparent K_m_^ADP^ of saponin-permeabilized rat cardiac fibers oxidizing palmitoyl-l-carnitine (9 μM) in the presence of malate (0.24 mM) or pyruvate + malate (control, 6 mM + 6 mM). Experiments were performed at 37 °C. * *p* < 0.05 vs. control. *n* = 5 (paired experiments). The results were analyzed with one-way analysis of variance (ANOVA) followed by Dunnett post hoc test.

The endogenous ADP is produced from exogenous ATP by ATPases in the myofibrils and in the sarcoplasmic reticulum, and is delivered directly to the mitochondria [8]. In the next series of experiments, the respiration of fibers was supported by palmitoyl-l-carnitine or pyruvate + malate (control), and titrated by ADP and ATP (Figure 8).

The apparent K_m_^ADP^ and K_m_^ATP^ were very low with palmitoyl-l-carnitine as the substrate (55 ± 9 and 59 ± 26 µM, respectively compared to pyruvate+malate (K_m_^ADP^, 312 ± 28 µM). Thus, in our study the decrease in the apparent K_m_^ADP^ during fatty acid oxidation did not depend on the exogenous or endogenous supply of ADP.

### 3.3. Influence of Palmitoyl-l-Carnitine on the Permeability of Mitochondrial Outer Membrane for ADP

**Figure 9:**
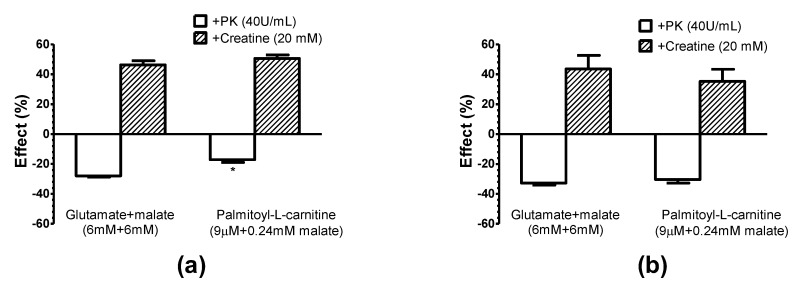
Influence of pyruvate kinase + phosphoenolpyruvate (PK + PEP) ADP-consuming system and creatine on the respiration of rat cardiac fibers using glutamate+malate and palmitoyl-l-carnitine + malate as respiratory substrates at (**a**) 25 °C and (**b**) 37 °C. The effect of pyruvate kinase (PK) (40 U/mL) on V_ATP_ and the effect of creatine on V_PK_ (40 U/mL) are shown. * *p* < 0.05 versus 37 °C with palmitoyl-l-carnitine, *n* = 5 (paired experiments). The results were analyzed with one-way analysis of variance (ANOVA) followed by Tukey’s multiple comparison test.

Using an exogenous ADP-trapping system consisting of pyruvate kinase + phosphoenolpyruvate (PK + PEP), which effectively competes with mitochondria for extramitochondrial ADP [8], we investigated the interactions of functional complexes of mitochondria with Ca, Mg ATPases of myofibrils and sarcoplasmic reticulum under different conditions (25 and 37 °C), and mitochondrial outer membrane permeability for ADP. The results of these experiments are presented in Figure 9. By the addition of 2 mM ATP we stimulated the basal respiration of mitochondria (similarly with both substrates, glutamate + malate and palmitoyl-l-carnitine), due to endogenously generated ADP. Consecutive addition of PK (40 U/mL, in the presence of PEP in the medium) suppressed respiration by about 30%; a similar effect (23%) was observed with palmitoyl-l-carnitine at 25 °C. The inhibitory effect of PK + PEP at 37 °C was equal with both respiratory substrates, glutamate + malate (33%) and palmitoyl-l-carnitine+malate (31%).

It is important to note that the exogenous cytochrome c test showed the intactness of the mitochondrial outer membrane (no stimulation of respiration in State 3 by cytochrome c was observed; its effect on respiration varied from 0.90 ± 0.02 to 1.04 ± 0.01; *n* = 5, paired experiments) and inaccessibility of PK to ADP localized in the intermembrane space of mitochondria. Thus, neither the increase in temperature (from 25 to 37 °C) nor fatty acid oxidation, which both largely increased the affinity of mitochondrial oxidative phosphorylation for exogenous and endogenous ADP (i.e., decrease in the apparent K_m_^ADP^ and K_m_^ATP^), affected the inhibitory effects of exogenous ADP-trapping system, and possibly, the concentration of ADP in the medium. In case palmitoyl-l-carnitine could increase the mitochondrial outer membrane permeability to ADP or ATP, the inhibitory effect of PK + PEP would have been different during palmitoyl-l-carnitine oxidation compared to the oxidation of pyruvate + malate. Our data show that the effect was similar during the oxidation of either substrate. Thus, the data presented above mean that the oxidation of palmitoyl-l-carnitine does not increase the mitochondrial outer membrane permeability to ADP or ATP.

### 3.4. Influence of Creatine Kinase on Regulation of Mitochondrial Respiration Supported by Fatty Acids

In the next series of experiments, we investigated the effect of fatty acid oxidation on the functional coupling between mitochondrial creatine kinase (mi-CK) and the ADP/ATP carrier.

The addition of 20 mM creatine in the presence of 2 mM ATP and exogenous PK + PEP system give notable and similar (1.35–1.56-fold) increase in the respiration rate in all conditions investigated (with both substrates, glutamate and palmitoyl-l-carnitine, at two different temperatures 25 and 37 °C, Figure 9).

Furthermore, the exogenous creatine, in the medium devoid of PK and PEP, stimulated the respiration of cardiac fibers oxidizing pyruvate + malate or palmitoyl-l-carnitine in presence of low 60 µM ADP concentration, accordingly by 1.37 ± 0.05 times and 1.42 ± 0.04 times (data of six unpaired experiments, 20 °C) with a similar efficacy as in the medium containing PK + PEP (Figure 9). It should be noted that in these experiments, exogenous cytochrome c had no significant stimulating effect on the mitochondrial respiration in State 3 with both substrates, indicating the intactness of the mitochondrial outer membrane.

In other series of experiments, fibers respiration supported by palmitoyl-l-carnitine was stimulated by exogenous ATP. In these conditions, endogenous ADP is produced from exogenous ATP by ATPases in myofibrils and the sarcoplasmic reticulum, and it is delivered directly to the mitochondria [13]. This approach should be regarded as more physiological. In this case the stimulating effect of creatine on mitochondrial respiration appeared to be very close to that observed in the above described experiments with the exogenous ADP; i.e., 1.38 ± 0.06 and 1.47 ± 0.05 times, respectively, with 23 and 37 μM of ATP (data of five unpaired experiments, 20 °C).

**Figure 10:**
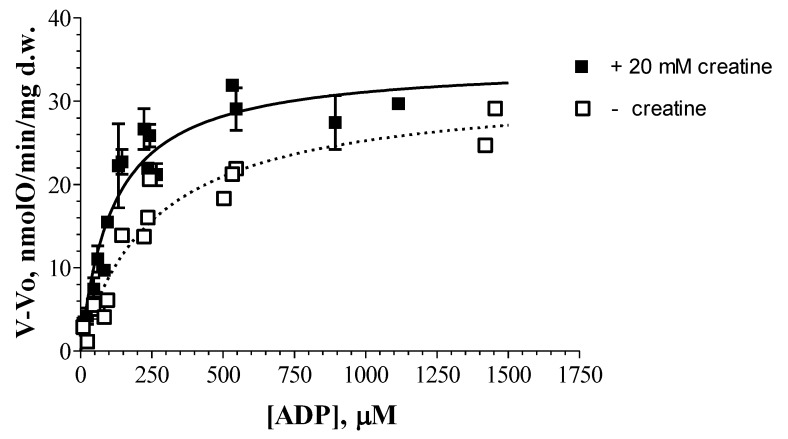
The dependence of the respiration rates of rat cardiac fibers on the external ADP concentration: the effect of creatine. The substrate: octanoyl-dl-carnitine 0.36 mM + malate 0.24 mM. The data of five separate paired experiments are presented. V_max_ values (in nmol O/min/mg dry weight) were found to be similar: 29.9 ± 1.8 (the medium without creatine) and 31.1 ± 1.6 (the medium with 20 mM creatine).

The maximal effect of creatine on mitochondrial respiration with octanoyl-dl-carnitine, similarly to palmitoyl-l-carnitine, was observed at 60 μM concentration of exogenous ADP (*n* = 3–5; Figure 10). Similar results (ADP concentration) were obtained also in the case of pyruvate + malate oxidation. When octanoyl-dl-carnitine was used as a respiratory substrate, the estimated apparent K_m_^ADP^ values were significantly different in the presence and absence of creatine, accordingly 98 ± 11 μM ADP and 235 ± 16 μM ADP (Figure 10).

Thus, the creatine-induced decrease in the apparent K_m_^ADP^ reflects the maintenance of functional coupling in the intermembrane space between ADP/ATP carrier and mi-CK in the mitochondria respiring on fatty acids.

### 3.5. Effects of Oncotic Pressure on Mitochondrial Fatty Acid Oxidation

**Figure 11:**
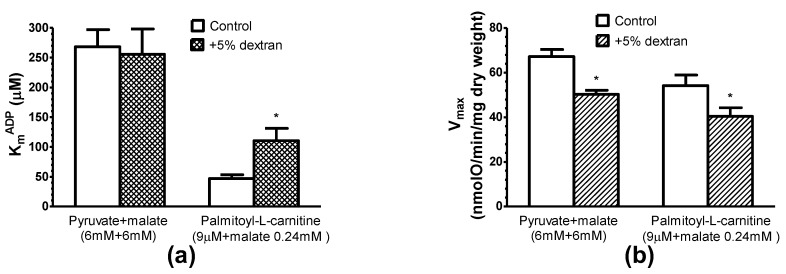
Influence of 5% dextran T70 on the apparent K_m_^ADP^ (**a**) and V_max_ (**b**) of saponin-permeabilized rat cardiac fibers using different respiratory substrates. *n* = 5 (paired), 37 °C, * *p* < 0.05 versus control. The results were analyzed with one-way analysis of variance (ANOVA) followed by a Dunnett post hoc test.

In our further study, 5% of dextran T-70 was added to the respiration medium to mimic the oncotic pressure of the cellular cytoplasm and to test its effect on the respiration of saponin-permeabilized rat cardiac fibers oxidizing pyruvate + malate or palmitoyl-l-carnitine. 5% dextran T-70 similarly (by 25%) decreased the maximal respiration rate (with ADP) of mitochondria in situ, oxidizing both substrates palmitoyl-l-carnitine and pyruvate + malate (Figure 11). Noteworthy, dextran did not affect the high apparent K_m_^ADP^ of mitochondria in cardiac fibers oxidizing pyruvate + malate, but significantly (by 50–60%) increased the low apparent K_m_^ADP^ of mitochondria oxidizing palmitoyl-l-carnitine. The apparent K_m_^ADP^ values during the oxidation of palmitoyl-l-carnitine in the medium containing 5% dextran (110.6 ± 20.7 µM) were much lower than the high apparent K_m_^ADP^ values (255.7 ± 42 µM) characteristic for the oxidation of pyruvate + malate.

### 3.6. Fatty Acid-Oxidation and Oncotic Pressure-induced Changes of Mitochondrial Morphology

**Figure 12:**
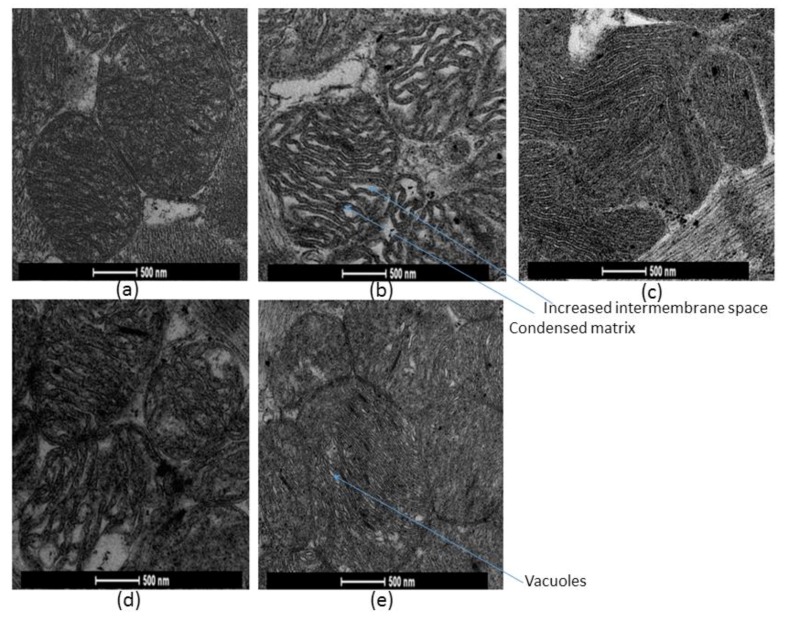
Morphology of mitochondria in skinned rat cardiac fibers. 5 min incubations of saponin-skinned cardiac fibers were performed in physiological salt solution at 37 °C in the presence of: (**a**) pyruvate + malate (6 mM + 6 mM); (**b**) palmitoyl-l-carnitine (9 µM); (**c**) palmitoyl-l-carnitine (9 µM) + 5% dextran T-70; (**d**) pyruvate + malate (6 mM + 6 mM) and ADP/ATP carrier inhibitor carboxyatractyloside (1.3 µM); (**e**) pyruvate + malate (6 mM + 6 mM) and ADP/ATP carrier inhibitor bongkrekic acid (17.6 µM).

Electron microscopy was used to evaluate morphological changes in mitochondria in situ caused by fatty acids oxidation in presence and absence of 5% dextran T-70 (Figure 12).

The results revealed that mitochondrial morphology in saponin-permeabilized cardiac fibers significantly alters during 5 min oxidation of palmitoyl-l-carnitine in the absence of dextran (Figure 12b); compared with pyruvate and malate oxidation (Figure 12a): increased mitochondrial intermembrane space and condensed mitochondrial matrix were observed.

In fibers treated with dextran (in the presence of palmitoyl-l-carnitine), the whole population of mitochondria was dark (Figure 12c) indicating that they all have intact outer membranes. This finding is in accordance with the functional evaluation of intactness of mitochondrial outer membrane by cytochrome c test. Dextran prevented the palmitoyl-l-carnitine-induced morphological changes of mitochondria, where condensed mitochondria were observed; i.e., the appearance of mitochondria became similar to that in fibers incubated with pyruvate and malate, or similar as with in vivo conditions.

It is noteworthy that the incubation of fibers with carboxyatractyloside (1.3 µM; at this concentration the state 3 respiration with pyruvate and malate was completely inhibited)-induced ultrastructural changes of mitochondria similar to those induced by palmitoyl-l-carnitine, corresponding to the orthodox state of mitochondria; that is, enlarged cristae volume, contracted matrix space (Figure 12d). Bongkrekic acid (17.6 µM) induced completely different changes of mitochondrial ultrastructure; mitochondria were dark, cristae compressed, matrix space enlarged, little vacuoles were noticed, corresponding to the condensed state of mitochondria (Figure 12e). Thus, our results suggest that fatty acid oxidation may cause the morphological changes of mitochondrial ultrastructure, with an orthodox conformation when the ADP/ATP carrier is in the cytosol-oriented state.

## 4. Discussion

Palmitoyl-l-carnitine and other fatty acids of different chain length and degree of saturation differently affect the various enzymes, carriers and functions of mitochondria [1,2,32]. Therefore, we investigated the role of decanoate, octanoyl-l-carnitine, palmitoyl-l-carnitine and oleoyl-CoA (plus carnitine) in the regulation of mitochondrial respiration. In contrast to oleoyl-CoA, short- and medium-chain fatty acids (up to twelve carbon atoms) can cross mitochondrial membranes bypassing the carnitine dependent transport system [33]. Decanoate is activated to acyl-CoA inside mitochondria before being directed to oxidation. This reaction is catalyzed by the medium-chain acyl-CoA synthetase and requires both CoA and ATP. Meanwhile, oleoyl-CoA does not need activation.

Despite of differences in transport pathways, some reactions preceding β-oxidation and the enzyme systems responsible for β-oxidation, decanoic acid and oleoyl-CoA, similarly decreased the apparent K_m_^ADP^ (Figure 2). Based on these findings and the related effects obtained previously with saturated fatty acids [9], it could be concluded that this property is general for all fatty acids. Furthermore, the decrease in the apparent K_m_^ADP^ was similar during palmitoyl-l-carnitine oxidation at concentrations resulting in the maximal State 3 respiration rate (9 µM) and the suboptimal one (at 2.2 µM and even at 0.5 μM; Figure 3). In addition, when pyruvate and malate were added immediately after the completion of the oxidation of a limited quantity of palmitoyl-l-carnitine (Figure 6), the apparent K_m_^ADP^ of the mitochondria in cardiac fibers remained at a high level, showing that the effect of fatty acids on the apparent K_m_^ADP^ of mitochondria in permeabilized rat cardiac fibers was reversible.

Levitsky and Skulachev [34] demonstrated that palmitoyl-l-carnitine, when transported into isolated rat liver mitochondria, induced a swelling of mitochondrial matrix. Neely and Feuvray [35] observed morphological changes of isolated heart mitochondria after incubation with palmitoyl-l-carnitine, and Piper et al. [36], with oleoyl-l-carnitine, and in addition, the surfactant properties of palmitoyl-l-carnitine were revealed (for review, see [37]). We examined in this study the possible influence of palmitoyl-l-carnitine on the swelling of respiring, isolated rat heart mitochondria and the intactness of their outer membrane. Our investigations showed clearly that the decrease of the apparent K_m_^ADP^ due to fatty acids oxidation described above cannot be attributed, neither to the increase in the volume of mitochondria (no mitochondrial swelling at 9 µM of palmitoyl-l-carnitine was observed; Figure 7), nor to the injury of mitochondrial outer membrane (no injury of mitochondrial outer membrane was demonstrated neither at 9 µM nor at much higher 80 µM of palmitoyl-l-carnitine, where large swelling was obvious; no injury of the mitochondrial outer membrane was also confirmed electron microscopically using dextran T-70; see Figure 12c and the discussion below).

Under some conditions (starvation, fasting) fatty acids play a major role in the heart energy metabolism. In the other cases, mostly both glucose and fatty acids are used as energy fuel, and as proposed [38], both are required for the optimal function of the failing heart; simultaneous oxidation of these two substrates is the best for heart function [39]. In this regard, our study shows the important effects of fatty acids on the regulation of the kinetics of oxidative phosphorylation (expressed as the apparent K_m_^ADP^ value) when they were used alone or in the combination with pyruvate and malate.

We compared the kinetics of the regulation of mitochondrial respiration stimulated both by the exogenous ADP and exogenous ATP. In the latter case, the endogenous ADP is produced by ATPases in the myofibrils and in the sarcoplasmic reticulum, and is delivered directly to the mitochondria [8]. In accordance with the low apparent K_m_^ADP^, also the low apparent K_m_^ATP^ values for saponin-permeabilized fibers respiring on palmitoyl-l-carnitine were demonstrated (for the first time with this substrate). In addition, our data are in accordance with the data obtained by Seppet et al. [8], where they demonstrated very similar high (300 μM) apparent K_m_ values for both exogenous ADP and exogenous ATP for cardiac fibers respiring on non-fatty substrates. Thus, the apparent K_m_ of oxidative phosphorylation for ADP in saponin-permeabilized rat cardiac fibers did not depend on the external or internal source of ADP.

When endogenous ADP is produced by ATPases in the myofibrils and in the sarcoplasmic reticulum, it is directly channeled to mitochondria without significant release into the medium if the mitochondrial oxidative phosphorylation is active [8]. This evidence was obtained by using an exogenous ADP-trapping system consisting of PK + PEP, which effectively competes with mitochondria for the extramitochondrial ADP, and therefore, decreases the respiration rate in the State 3. In our experiments, the PK effect is exceptionally from the outside of the mitochondria, since the exogenous cytochrome c test (no stimulation of respiration in State 3 by cytochrome c) shows the intactness of the mitochondrial outer membrane and the inaccessibility of PK to ADP localized in the intermembrane space of the mitochondria. Thus, neither the increase in temperature (from 25 to 37 °C), nor fatty acid oxidation, which both largely increase the affinity of mitochondrial oxidative phosphorylation for exogenous and endogenous ADP (decrease in the apparent K_m_^ADP^ and apparent K_m_^ATP^), affect the inhibitory effects of the exogenous ADP-trapping system (Figure 9), and thus did not enhance the permeability of the mitochondrial outer membrane for ADP (Figure 9). The inhibitory effects of PK + PEP on the respiration of saponin-permeabilized rat cardiac fibers oxidizing glutamate+malate (25 °C) obtained by us are in good agreement with the data of other investigators [8,10].

The stimulating effects of creatine on fibers respiration with palmitoyl-l-carnitine assessed at low concentration (60 μM) of ADP reflects maintenance of functional coupling in the intermembrane space between the ADP/ATP carrier and mi-CK. Our data (Figure 10) show that the functional coupling between the ADP/ATP carrier and mi-CK is preserved in mitochondria despite the significant decrease in the apparent K_m_^ADP^ induced by fatty acid oxidation. Noteworthy, the latter phenomenon is observed not only at 37 °C [9], but also at lower, i.e., 20 °C temperature. The finding that creatine significantly decreases the apparent K_m_^ADP^ in the mitochondria oxidizing octanoyl-dl-carnitine (Figure 5) is in a good agreement with the data of other investigators [40,41], when pyruvate+malate, i.e., non-fatty respiratory substrate, was used. 

There are two mechanisms suggested to explain the effective interaction between mi-CK and the ADP/ATP carrier: the dynamic compartmentation of ATP and ADP [42], and the direct transfer of ATP and ADP between the proteins [43,44]. According to the first mechanism, the functional coupling between mi-CK and the ADP/ATP carrier can be explained by differences between the concentrations of ATP and ADP in the intermembrane space and those in the surrounding solution due to some limitations of their diffusion across the outer mitochondrial membrane [42]. Beside dynamic compartmentation, facilitated diffusion was also suggested as the potential mechanism of the action of the phosphocreatine shuttle [45]. According to the second mechanism of coupling, ATP and ADP are directly transferred between mi-CK and the ADP/ATP carrier without leaving the complex of proteins [44]. Kuznetsov et al. [41] investigated the apparent Km for ADP in cardiac, slow-twitch and fast-twitch skeletal muscle fibers, and they provide a strong argument against the possibility that the diffusion problems may be related to the increased apparent K_m_ for ADP in cardiac and slow-twitch skeletal muscle fibers, as in fast-twitch skeletal muscle fibers, it remains low and comparable to the apparent K_m_ for ADP in isolated mitochondria [41]. Also, they found that in the ghost fibers where the myosin and 10–20% of cellular proteins were removed from the cells by KC1 treatment, the apparent K_m_ for ADP remained unchanged [41]. Under their conditions the sarcolemma was almost completely removed, and could not act as a diffusion barrier even for the big protein molecules [41]. Their data together with our results that creatine did not increase V_max_ in skinned cardiac fibers oxidizing octanoyl-dl-carnitine (Figure 10), and that the addition of dextran did not affect the apparent K_m_ for ADP in the skinned cardiac fibers oxidizing pyruvate and malate (Figure 11a), suggest that the changes in the apparent K_m_ for ADP are unlikely related to the diffusion problems.

In vivo mitochondria are embedded in the cytoplasm in which the protein content may be as high as 20–30% (*m*/*v*) [46]. Macromolecules, like bovine serum albumin, dextran, ficol, polyvinylpyrrolidone, added to the medium, can restore the morphological changes in the outer mitochondrial compartment that occur during the isolation of mitochondria [47,48,49]. 

It has been also shown that, in isolated rat heart mitochondria, 10% of bovine serum albumin and 5–25% dextran strongly increase the apparent K_m_^ADP^ of oxidative phosphorylation [15,50] and of mitochondrial creatine kinase [50]. Dextrans or other macromolecules decrease the conductivity of porin pores in artificial membranes [48,51], the volume of the intermembrane space in isolated mitochondria, and increase the number of contact sites between both mitochondrial membranes [52]. Morphological changes of isolated mitochondria are accompanied by a reduced permeability of the mitochondrial outer membrane for adenine nucleotides [50]. In this study, respirometric investigation of saponin-permeabilized rat cardiac fibers demonstrated that an addition of 5% dextran into the incubation medium (to mimic the oncotic pressure of the cellular cytoplasm) markedly increased the low apparent K_m_^ADP^ value of mitochondria respiring on palmitoyl-l-carnitine, but did not affect the high apparent K_m_^ADP^ of mitochondria respiring on pyruvate and malate (Figure 11). Interestingly, the apparent K_m_^ADP^ values during the oxidation of palmitoyl-l-carnitine in the medium containing 5% dextran (110.6 ± 20.7 μM ADP), despite some increase (compared with medium devoid of dextran), remained far below high values characteristic for the oxidation of pyruvate + malate (apparent K_m_^ADP^: 255.7 ± 42 µM) (Figure 11).

Electron microscopy was used to evaluate the effects of fatty acid oxidation and dextran T-70 on the morphology of rat heart mitochondria in situ (Figure 12). Palmitoyl-l-carnitine oxidation induced marked alterations in the mitochondrial ultrastructure, which is different from that observed with pyruvate and malate, and similar to the changes induced by the incubation of fibers with the ADP/ATP carrier inhibitor carboxyatractyloside. When mitochondria were incubated with compounds, fixing the ADP/ATP carrier in M (i.e., matrix-oriented) conformation, the cristae were compressed, the matrix space enlarged, and the separation of the inner membrane space forming vacuoles was noticed [53]. We obtained similar results after the incubation of mitochondria with bongkrekic acid. In the contrary, when mitochondria were incubated with compounds, fixing the ADP/ATP carrier in C (i.e., cytosol-oriented) conformation, e.g., with carboxyatractyloside, such that the cristae volume was enlarged at the expense of matrix space, the electron densities increased [53]. Furthermore, these two distinct mitochondrial conformations corresponded to condensed and orthodox mitochondrial conformation, accordingly [25,26]. Ultrastructural changes of mitochondria in cardiac fibers respiring on palmitoyl-l-carnitine were completely prevented by dextran addition into the incubation medium. Furthermore, most mitochondria in the fibers incubated with palmitoyl-l-carnitine in the presence of dextran appeared dark, indicating that their outer membranes were intact (Figure 12c), in accordance with the results of the cytochrome c test, presented in the Results section. Partial “normalization”, i.e., the increase by dextran of the apparent K_m_^ADP^ and complete “normalization” of the ultrastructure of mitochondria in saponin-permeabilized cardiac fibers respiring on palmitoyl-l-carnitine could be due to fatty acid oxidation-induced alterations of the mitochondrial ultrastructure.

The high apparent K_m_ for exogenous ADP is determined by low mitochondrial outer membrane permeability for ADP and the concerted action of tight complexes (called as intracellular energetic units) of mitochondria and other cellular ADP-producing systems (ATPases) in myofibrils and sarcoplasmic reticulum [8,10]. A highly organized intracellular structure and the arrangement of mitochondria are also crucial for the increase in the affinity of mitochondrial oxidative phosphorylation for ADP and the decrease in the apparent K_m_^ADP^ value [16,18,22]. 

The calculations by Lizana et al. [54] using a simplistic model suggested that mitochondria (and small biological compartments in general) may regulate the dynamics of interior reaction pathways (e.g., the Krebs cycle) by volume changes. It was hypothesized (for review, see [55]) that the cristae shape can be modulated by different pathways/signaling molecules (ROS, the NADH/NAD ratio, the ATP/ADP ratio, etc.). Even low concentration of long-chain fatty acid palmitoyl-l-carnitine oxidation in skeletal muscle and heart mitochondria has been associated with significantly higher ROS production and increased mitochondrial proton leak (2 times lower respiratory control ratio, i.e., coupling), compared with the oxidation of NADH-linked substrates (pyruvate + malate or glutamate + malate) [56,57,58]. 

The greater ROS formation, similar at higher and lower mitochondrial membrane potential, was maintained despite of the activation of the uncoupling mechanisms of the ADP/ATP carrier during palmitoyl-l-carnitine (18 µM) oxidation [56]. Furthermore, functional (and possibly structural) interaction of ADP/ATP carrier and VDAC and its modulation/interruption by the ADP/ATP carrier inhibitor atractyloside-induced change of ADP/ATP carrier conformation has been also demonstrated [59]. Structural changes of mitochondria induced by carboxyatractyloside, similar to those induced by palmitoyl-l-carnitine, were observed by us in this study. The functional interaction of ADP/ATP carrier and VDAC facilitated the channeling of nucleotides and other metabolites between cytosol and mitochondrial matrix [59].

The low apparent K_m_^ADP^ in mitochondria from cancerous cells [22,23] and fetal or neonatal mitochondria [24] could be related to mitochondrial ultrastructural changes. Adult mitochondria are usually in condensed conformation, with enlarged mitochondrial matrix volume and the decreased intermembrane space, compared to neonatal mitochondria which are mostly in orthodox conformation [25,26]. Our results showed that palmitoyl-l-carnitine oxidation induced ultrastructural changes of mitochondria, with a marked increase in mitochondrial intermembrane space and decrease of mitochondrial matrix (Figure 12b), and similar changes were obtained in the presence of the ADP/ATP carrier inhibitor carboxyatractyloside (Figure 12d).

Recent studies have revealed that fatty acids could be the cofactors [60] of the newly discovered action mode of the ADP/ATP carrier when protons are transported to mitochondrial matrix [61]. Cytosolic fatty acids could interfere with ADP/ATP transport [60] by activating the proton current to mitochondrial matrix [61]. They are able to bind to the ADP/ATP carrier from the cytosolic side in the C- or M-state, but could not induce C–M conformational change or be transported by the ADP/ATP carrier [61]. It was suggested that the ADP/ATP carrier could have two transport modes: C–M conformational change-related electrogenic ADP/ATP exchange and cytosolic fatty acid-activated conformation-independent proton channel [61]. We have also observed that palmitate, palmitoyl-CoA and palmitoyl-l-carnitine could not induce the decrease of apparent K_m_^ADP^ when their oxidation was prevented by the absence of necessary cofactors or blocked with rotenone [9]. The lower apparent K_m_^ADP^ was related to the oxidation, but not to the transport of fatty acids into mitochondria [9]. In the study of Divakaruni et al. [19], fatty acids have been demonstrated to change the conformation of the uncoupling protein, that is structurally, and to some extent, functionally, similar with the ADP/ATP carrier [20,21]. ADP/ATP exchange is driven by the mitochondrial membrane potential, which has been shown to affect the distribution of the binding sites of the ADP/ATP carrier between inside and outside, as well as the distribution of ADP/ATP carrier molecules between the M- and the C-state, respectively [62]. Thus, Krämer and Klingenberg argued that the membrane potential could indirectly influence the C- and M-conformational transition of the ADP/ATP carrier [62]. The tumor and fetal/neonatal mitochondria are not only characterized by the low apparent K_m_ ADP, but also have higher mitochondrial membrane potential, i.e., are hyperpolarized [63,64]. According to the data of our colleagues, the mitochondrial membrane potential of isolated rat heart mitochondria respiring on pyruvate+malate or palmitoyl-l-carnitine as substrates was accordingly 146 ± 2 mV and 124 ± 1 mV (*n* = 5, assessed with TPP^+^ electrode; R. Baniene, unpublished data, 2020); i.e., the membrane potential did not increase, rather it slightly decreased during palmitoyl-l-carnitine oxidation. These values are in line with the data from the study of Seifert et al. where lower energization and lower membrane potential of the skeletal muscle mitochondria were reported in the case of palmitoyl-l-carnitine oxidation compared to pyruvate and malate oxidation [56]; however, it could be related to the higher uncoupling protein content in skeletal muscle mitochondria [65]. It is technically complicated to quantify correctly the mitochondrial membrane potential in skinned cardiac fibers due to the distribution of the cationic potential probes within the cellular structures present in this object. However, it could not be excluded that the mitochondrial membrane potential in the skinned fibers might differ from the mitochondrial potential in the isolated mitochondria. 

Thus, the hypothesis that the fatty acid oxidation-induced conformational change of the ADP/ATP carrier (M-state to C-state, condensed to orthodox mitochondria) affecting the oxidative phosphorylation affinity for ADP could be driven by the higher membrane potential generated during fatty acid oxidation might be an interesting question to address in the future research.

Overall, our results imply that the fatty acid oxidation could regulate cellular energy metabolism by increasing oxidative phosphorylation affinity for ADP due to fatty acid oxidation-induced ADP/ATP carrier switch from M- to C-state, and corresponding mitochondrial transition from condensed to orthodox conformation. Furthermore, this mechanism could be responsible for the altered mitochondrial metabolism [66] when fatty acid oxidation is increased during the development of chronic [67,68] and age-associated disorders, such as cardiovascular diseases, diabetes, neurodegenerative diseases and cancer [69,70], and the ADP/ATP carrier modulation could be a promising target in the search of novel therapies to restore the normal mitochondrial function.

## References

[1] Vignais P.V. (1976). Molecular and physiological aspects of adenine nucleotide transport in mitochondria. Biochim. Biophys. Acta.

[2] Wojtczak L. (1976). Effect of long-chain fatty acids and acyl-CoA on mitochondrial permeability, transport, and energy-coupling processes. J. Bioenerg. Biomembr..

[3] Qu Q., Zeng F., Liu X., Wang Q.J., Deng F. (2016). Fatty acid oxidation and carnitine palmitoyltransferase I: Emerging therapeutic targets in cancer. Cell Death Dis..

[4] Roussel J., Thireau J., Brenner C., Saint N., Scheuermann V., Lacampagne A., Le Guennec J.Y., Fauconnier J. (2015). Palmitoyl-carnitine increases RyR2 oxidation and sarcoplasmic reticulum Ca2+ leak in cardiomyocytes: Role of adenine nucleotide translocase. Biochim. Biophys. Acta.

[5] Lopaschuk G.D., Ussher J.R., Folmes C.D., Jaswal J.S., Stanley W.C. (2010). Myocardial fatty acid metabolism in health and disease. Physiol. Rev..

[6] Klingenberg M. (2008). The ADP and ATP transport in mitochondria and its carrier. Biochim. Biophys. Acta.

[7] Saks V.A., Khuchua Z.A., Vasilyeva E.V., Belikova O., Kuznetsov A.V. (1994). Metabolic compartmentation and substrate channelling in muscle cells. Role of coupled creatine kinases in in vivo regulation of cellular respiration—A synthesis. Mol. Cell Biochem..

[8] Seppet E.K., Kaambre T., Sikk P., Tiivel T., Vija H., Tonkonogi M., Sahlin K., Kay L., Appaix F., Braun U. (2001). Functional complexes of mitochondria with Ca,MgATPases of myofibrils and sarcoplasmic reticulum in muscle cells. Biochim. Biophys. Acta.

[9] Toleikis A., Liobikas J., Trumbeckaite S., Majiene D. (2001). Relevance of fatty acid oxidation in regulation of the outer mitochondrial membrane permeability for ADP. FEBS Lett..

[10] Saks V.A., Kaambre T., Sikk P., Eimre M., Orlova E., Paju K., Piirsoo A., Appaix F., Kay L., Regitz-Zagrosek V. (2001). Intracellular energetic units in red muscle cells. Biochem. J..

[11] Saks V., Kuznetsov A.V., Gonzalez-Granillo M., Tepp K., Timohhina N., Karu-Varikmaa M., Kaambre T., Dos Santos P., Boucher F., Guzun R. (2012). Intracellular Energetic Units regulate metabolism in cardiac cells. J. Mol. Cell Cardiol..

[12] Dzeja P.P., Hoyer K., Tian R., Zhang S., Nemutlu E., Spindler M., Ingwall J.S. (2011). Rearrangement of energetic and substrate utilization networks compensate for chronic myocardial creatine kinase deficiency. J. Physiol..

[13] Kaasik A., Veksler V., Boehm E., Novotova M., Minajeva A., Ventura-Clapier R. (2001). Energetic crosstalk between organelles: Architectural integration of energy production and utilization. Circ. Res..

[14] Toleikis A., Majiene D., Trumbeckaite S., Dagys A., Jasaitis A. (1996). The effect of collagenase and temperature on mitochondrial respiratory parameters in saponin-skinned cardiac fibers. Biosci. Rep..

[15] Liobikas J., Kopustinskiene D.M., Toleikis A. (2001). What controls the outer mitochondrial membrane permeability for ADP: Facts for and against the role of oncotic pressure. Biochim. Biophys. Acta.

[16] Appaix F., Kuznetsov A.V., Usson Y., Kay L., Andrienko T., Olivares J., Kaambre T., Sikk P., Margreiter R., Saks V. (2003). Possible role of cytoskeleton in intracellular arrangement and regulation of mitochondria. Exp. Physiol..

[17] Guzun R., Timohhina N., Tepp K., Monge C., Kaambre T., Sikk P., Kuznetsov A.V., Pison C., Saks V. (2009). Regulation of respiration controlled by mitochondrial creatine kinase in permeabilized cardiac cells in situ. Importance of system level properties. Biochim. Biophys. Acta.

[18] Boudina S., Laclau M.N., Tariosse L., Daret D., Gouverneur G., Bonoron-Adele S., Saks V.A., Dos Santos P. (2002). Alteration of mitochondrial function in a model of chronic ischemia in vivo in rat heart. Am. J. Physiol. Heart Circ. Physiol..

[19] Divakaruni A.S., Humphrey D.M., Brand M.D. (2012). Fatty acids change the conformation of uncoupling protein 1 (UCP1). J. Biol. Chem..

[20] Aquila H., Link T.A., Klingenberg M. (1985). The uncoupling protein from brown fat mitochondria is related to the mitochondrial ADP/ATP carrier. Analysis of sequence homologies and of folding of the protein in the membrane. EMBO J..

[21] Klingenberg M. (1990). Mechanism and evolution of the uncoupling protein of brown adipose tissue. Trends Biochem. Sci..

[22] Anmann T., Guzun R., Beraud N., Pelloux S., Kuznetsov A.V., Kogerman L., Kaambre T., Sikk P., Paju K., Peet N. (2006). Different kinetics of the regulation of respiration in permeabilized cardiomyocytes and in HL-1 cardiac cells. Importance of cell structure/organization for respiration regulation. Biochim. Biophys. Acta.

[23] Eimre M., Paju K., Pelloux S., Beraud N., Roosimaa M., Kadaja L., Gruno M., Peet N., Orlova E., Remmelkoor R. (2008). Distinct organization of energy metabolism in HL-1 cardiac cell line and cardiomyocytes. Biochim. Biophys. Acta.

[24] Anmann T., Varikmaa M., Timohhina N., Tepp K., Shevchuk I., Chekulayev V., Saks V., Kaambre T. (2014). Formation of highly organized intracellular structure and energy metabolism in cardiac muscle cells during postnatal development of rat heart. Biochim. Biophys. Acta.

[25] Valcarce C., Navarrete R.M., Encabo P., Loeches E., Satrustegui J., Cuezva J.M. (1988). Postnatal development of rat liver mitochondrial functions. The roles of protein synthesis and of adenine nucleotides. J. Biol. Chem..

[26] Valcarce C., Cuezva J.M. (1991). Interaction of adenine nucleotides with the adenine nucleotide translocase regulates the developmental changes in proton conductance of the inner mitochondrial membrane. FEBS Lett..

[27] Benard G., Faustin B., Passerieux E., Galinier A., Rocher C., Bellance N., Delage J.P., Casteilla L., Letellier T., Rossignol R. (2006). Physiological diversity of mitochondrial oxidative phosphorylation. Am. J. Physiol. Cell Physiol..

[28] Fernandez-Vizarra E., Enriquez J.A., Perez-Martos A., Montoya J., Fernandez-Silva P. (2011). Tissue-specific differences in mitochondrial activity and biogenesis. Mitochondrion.

[29] Kuznetsov A.V., Veksler V., Gellerich F.N., Saks V., Margreiter R., Kunz W.S. (2008). Analysis of mitochondrial function in situ in permeabilized muscle fibers, tissues and cells. Nat. Protoc..

[30] Holtzman J.L. (1976). Calibration of the oxygen polarograph by the depletion of oxygen with hypoxanthine-xanthine oxidase-catalase. Anal. Chem..

[31] Gornall A.G., Bardawill C.J., David M.M. (1949). Determination of serum proteins by means of the biuret reaction. J. Biol. Chem..

[32] Sultan A., Sokolove P.M. (2001). Free fatty acid effects on mitochondrial permeability: An overview. Arch. Biochem. Biophys..

[33] Kerner J., Hoppel C. (2000). Fatty acid import into mitochondria. Biochim. Biophys. Acta.

[34] Levitsky D.O., Skulachev V.P. (1972). Carnitine: The carrier transporting fatty acyls into mitochondria by means of an electrochemical gradient of H+. Biochim. Biophys. Acta.

[35] Neely J.R., Feuvray D. (1981). Metabolic products and myocardial ischemia. Am. J. Pathol..

[36] Piper M.H., Sezer O., Schwartz P., Hutter J.F., Schweickhardt C., Spieckermann P.G. (1984). Acyl-carnitine effects on isolated cardiac mitochondria and erythrocytes. Basic Res. Cardiol..

[37] Goni F.M., Requero M.A., Alonso A. (1996). Palmitoylcarnitine, a surface-active metabolite. FEBS Lett..

[38] Tuunanen H., Engblom E., Naum A., Nagren K., Hesse B., Airaksinen K.E., Nuutila P., Iozzo P., Ukkonen H., Opie L.H. (2006). Free fatty acid depletion acutely decreases cardiac work and efficiency in cardiomyopathic heart failure. Circulation.

[39] Taegtmeyer H. (2000). Metabolism--the lost child of cardiology. J. Am. Coll. Cardiol..

[40] Laclau M.N., Boudina S., Thambo J.B., Tariosse L., Gouverneur G., Bonoron-Adele S., Saks V.A., Garlid K.D., Dos Santos P. (2001). Cardioprotection by ischemic preconditioning preserves mitochondrial function and functional coupling between adenine nucleotide translocase and creatine kinase. J. Mol. Cell Cardiol..

[41] Kuznetsov A.V., Tiivel T., Sikk P., Kaambre T., Kay L., Daneshrad Z., Rossi A., Kadaja L., Peet N., Seppet E. (1996). Striking differences between the kinetics of regulation of respiration by ADP in slow-twitch and fast-twitch muscles in vivo. Eur. J. Biochem..

[42] Gellerich F.N., Schlame M., Bohnensack R., Kunz W. (1987). Dynamic compartmentation of adenine nucleotides in the mitochondrial intermembrane space of rat-heart mitochondria. Biochim. Biophys. Acta.

[43] Saks V.A., Chernousova G.B., Gukovsky D.E., Smirnov V.N., Chazov E.I. (1975). Studies of energy transport in heart cells. Mitochondrial isoenzyme of creatine phosphokinase: Kinetic properties and regulatory action of Mg2+ ions. Eur. J. Biochem..

[44] Jacobus W.E., Saks V.A. (1982). Creatine kinase of heart mitochondria: Changes in its kinetic properties induced by coupling to oxidative phosphorylation. Arch. Biochem. Biophys..

[45] Meyer R.A., Sweeney H.L., Kushmerick M.J. (1984). A simple analysis of the “phosphocreatine shuttle”. Am. J. Physiol..

[46] Fulton A.B. (1982). How crowded is the cytoplasm?. Cell.

[47] Bakeeva L.E., Chentsov Y.S., Jasaitis A.A., Skulachev V.P. (1972). The effect of oncotic pressure on heart muscle mitochondria. Biochim. Biophys. Acta.

[48] Wicker U., Bucheler K., Gellerich F.N., Wagner M., Kapischke M., Brdiczka D. (1993). Effect of macromolecules on the structure of the mitochondrial inter-membrane space and the regulation of hexokinase. Biochim. Biophys. Acta.

[49] Wrogemann K., Nylen E.G., Adamson I., Pande S.V. (1985). Functional studies on in situ-like mitochondria isolated in the presence of polyvinyl pyrrolidone. Biochim. Biophys. Acta.

[50] Gellerich F.N., Laterveer F.D., Korzeniewski B., Zierz S., Nicolay K. (1998). Dextran strongly increases the Michaelis constants of oxidative phosphorylation and of mitochondrial creatine kinase in heart mitochondria. Eur. J. Biochem..

[51] Gellerich F.N., Wagner M., Kapischke M., Wicker U., Brdiczka D. (1993). Effect of macromolecules on the regulation of the mitochondrial outer membrane pore and the activity of adenylate kinase in the inter-membrane space. Biochim. Biophys. Acta.

[52] Gellerich F.N., Kapischke M., Kunz W., Neumann W., Kuznetsov A., Brdiczka D., Nicolay K. (1994). The influence of the cytosolic oncotic pressure on the permeability of the mitochondrial outer membrane for ADP: Implications for the kinetic properties of mitochondrial creatine kinase and for ADP channelling into the intermembrane space. Mol. Cell Biochem..

[53] Nohl H. (1982). Age-dependent changes in the structure-function correlation of ADP/ATP-translocating mitochondrial membranes. Gerontology.

[54] Lizana L., Bauer B., Orwar O. (2008). Controlling the rates of biochemical reactions and signaling networks by shape and volume changes. Proc. Natl. Acad. Sci. USA.

[55] Cogliati S., Enriquez J.A., Scorrano L. (2016). Mitochondrial Cristae: Where Beauty Meets Functionality. Trends Biochem. Sci..

[56] Seifert E.L., Estey C., Xuan J.Y., Harper M.E. (2010). Electron transport chain-dependent and -independent mechanisms of mitochondrial H2O2 emission during long-chain fatty acid oxidation. J. Biol. Chem..

[57] St-Pierre J., Buckingham J.A., Roebuck S.J., Brand M.D. (2002). Topology of superoxide production from different sites in the mitochondrial electron transport chain. J. Biol. Chem..

[58] Liesa M., Shirihai O.S. (2013). Mitochondrial dynamics in the regulation of nutrient utilization and energy expenditure. Cell Metab..

[59] Allouche M., Pertuiset C., Robert J.L., Martel C., Veneziano R., Henry C., dein O.S., Saint N., Brenner C., Chopineau J. (2012). ANT-VDAC1 interaction is direct and depends on ANT isoform conformation in vitro. Biochem. Biophys. Res. Commun..

[60] Bernardi P. (2019). Mitochondrial H+ permeability through the ADP/ATP carrier. Nat. Metab..

[61] Bertholet A.M., Chouchani E.T., Kazak L., Angelin A., Fedorenko A., Long J.Z., Vidoni S., Garrity R., Cho J., Terada N. (2019). H(+) transport is an integral function of the mitochondrial ADP/ATP carrier. Nature.

[62] Kramer R., Klingenberg M. (1982). Electrophoretic control of reconstituted adenine nucleotide translocation. Biochemistry.

[63] Heerdt B.G., Houston M.A., Augenlicht L.H. (2006). Growth properties of colonic tumor cells are a function of the intrinsic mitochondrial membrane potential. Cancer Res..

[64] Chung S., Dzeja P.P., Faustino R.S., Perez-Terzic C., Behfar A., Terzic A. (2007). Mitochondrial oxidative metabolism is required for the cardiac differentiation of stem cells. Nat. Clin. Pr. Cardiovasc. Med..

[65] Ricquier D. (1999). Mitochondrial uncoupling proteins. Curr. Opin. Drug Discov. Devel..

[66] Porporato P.E., Filigheddu N., Pedro J.M.B., Kroemer G., Galluzzi L. (2018). Mitochondrial metabolism and cancer. Cell Res..

[67] Shammas M.A., Neri P., Koley H., Batchu R.B., Bertheau R.C., Munshi V., Prabhala R., Fulciniti M., Tai Y.T., Treon S.P. (2006). Specific killing of multiple myeloma cells by (-)-epigallocatechin-3-gallate extracted from green tea: Biologic activity and therapeutic implications. Blood.

[68] Braicu C., Ladomery M.R., Chedea V.S., Irimie A., Berindan-Neagoe I. (2013). The relationship between the structure and biological actions of green tea catechins. Food Chem..

[69] Majumder K., Mine Y., Wu J. (2016). The potential of food protein-derived anti-inflammatory peptides against various chronic inflammatory diseases. J. Sci. Food Agric..

[70] Zhang Y.J., Gan R.Y., Li S., Zhou Y., Li A.N., Xu D.P., Li H.B. (2015). Antioxidant Phytochemicals for the Prevention and Treatment of Chronic Diseases. Molecules.

